# Anisotropic Generation and Detection of Coherent A_g_ Phonons in Black Phosphorus

**DOI:** 10.3390/nano11051202

**Published:** 2021-05-01

**Authors:** Seong-Yeon Lee, Ki-Ju Yee

**Affiliations:** Department of Physics and Institute of Quantum Systems, Chungnam National University, Daejeon 34134, Korea; lsy929292@naver.com

**Keywords:** black phosphorus, optical anisotropy, coherent A_g_ phonons

## Abstract

Black phosphorus (BP) has attracted great attention due to its layer-tuned direct bandgap, in-plane anisotropic properties, and novel optoelectronic applications. In this work, the anisotropic characteristics of BP crystal in terms of the Raman tensor and birefringence are studied by investigating polarization dependence in both the generation and detection of A_g_ mode coherent phonons. While the generated coherent phonons exhibit the typical linear dichroism of BP crystal, the detection process is found here to be influenced by anisotropic multiple thin film interference, showing wavelength and sample thickness sensitive behaviors. We additionally find that the A_g_^1^ and A_g_^2^ optical phonons decay into lower frequency acoustic phonons through the temperature-dependent anharmonic process.

## 1. Introduction

Since the demonstration of its in-plane anisotropy in 2014, black phosphorus (BP) is gaining great attention for its unique physical properties and potential applications [1,2,3,4,5,6,7]. The anisotropic (orientation sensitive) characteristics of the electronic, optoelectronic, thermal, and mechanical properties of BP have motivated extensive research activities, leading to the development of chiral-selective novel devices such as polarization sensitive photodetectors and neuromorphic synapse applications [8,9,10,11]. Moreover, BP layers exhibit a layer number dependent systematic tuning of the direct energy gap, from 2.2 eV for monolayer to 0.3 eV for bulk BP [12], which is useful in optoelectronic device applications.

The large in-plane anisotropy of various BP properties is attributed to its puckered crystal structure, consisting of two layers of group V phosphorous atoms. In the optical response, the absorption coefficient and the refractive index are strongly dependent on the crystal direction of BP [13,14]. Anisotropies in the electrical transport and thermal conductivity of BP have also been demonstrated [1,15,16,17]. Additionally, the anisotropic electron–phonon interaction in BP leads to strongly polarization-dependent Raman scattering behaviors [18,19,20,21,22,23,24,25], where sensitive changes by layer thickness were explained by the interference effect of either the incident or the scattered light [18]. Recently, Raman scattering with an orthogonal polarization configuration and the B_2g_ Raman mode under a nonanalyzer configuration were demonstrated to be useful in identifying the crystalline orientation of BP [26,27].

While inelastic light scattering in the frequency domain is measured via Raman scattering, electron–phonon interactions can be investigated in the time domain by generating and detecting coherent phonons (CPs) with ultrashort optical pulses [28,29,30,31,32]. Upon electronic perturbation by an ultrashort pulse, the lattice is driven to macroscopic motions, thereby generating CPs of specific modes. If optical properties are modulated by lattice vibrations, the lattice motion can be visualized by measuring the transmittance or reflectance in the time domain. In studying CPs with such a pump-probe method, in addition to adjusting the pump polarization in the generation process, the probe polarization can also be adjusted in the detection process. Furthermore, we note that the CP signals, by visualizing the crystal vibration in the time domain, reveal the dynamics and phase information of the lattice motion.

In this paper, by performing time-resolved pump-probe experiments with ultrashort pulses, we report on the anisotropic generation and detection of coherent A_g_^1^ and A_g_^2^ phonons in multilayer BP crystal. The polarization dependence of the coherent A_g_ phonon generation, being maximum at the armchair (AC) polarization, follows the same anisotropic behavior as from the linear dichroism of BP. On the other hand, a rather complicated probe polarization dependence, varying with sample thickness and laser wavelength, is found to originate from interference in the CP signal from multiple reflections at the front and back interfaces of the BP. In addition, we use the anharmonic phonon decay model to explain the temperature-dependent decay rate and frequency shift of the A_g_ phonons.

## 2. Materials and Methods

**Figure 1:**
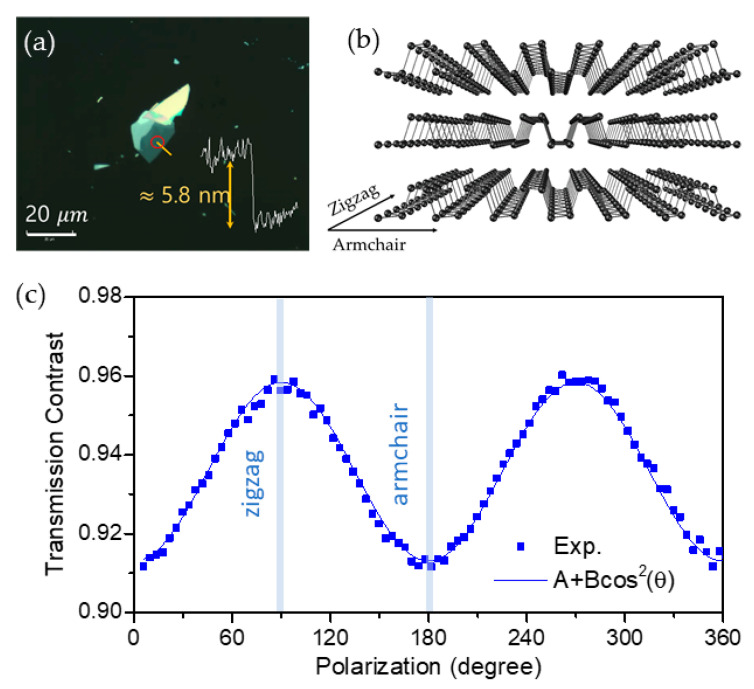
(**a**) Optical microscope image and AFM profile of the 5.8 nm thick BP flake. The area at which pump-probe measurements were performed is marked with a red circle. (**b**) Schematic diagram of the crystal structure of multilayer BP. (**c**) Polarization-resolved transmission showing the linear dichroism of BP. The solid line is a fitting with the A + B × cos^2^(*θ*) function.

We mechanically exfoliated multilayer BP flakes using the so-called scotch tape method [2,3] from a bulk BP manufactured by HQ Graphene. When an adhesive tape is attached to and then removed from a bulk BP crystal, BP flakes are randomly separated and stick to the tape. After repeated peelings, we attached and detached the tape containing numerous flakes onto a fused silica window. A large number of BP flakes of varying thickness and size were eventually produced on the fused silica substrate. Through optical microscope inspection, we selected several clean and large-area BP flakes for further studies. In order to confirm the thickness of each flake, we measured the surface topology with contact mode scanning of an atomic force microscope (AFM) (model XE-120, Park Systems). Figure 1a shows an optical image of one of the BP flakes (sample A) in this study, which was obtained with a total magnification of ×1000 using a microscope system (model MX61, Olympus). The bottom area within the flake had a thickness of about 5.8 nm, and we performed pump-probe experiments on the spot marked by a red circle in Figure 1a. As shown in Figure 1b, the crystal structure of BP has two principal axes, of which the AC axis is along the puckered direction and the zigzag (ZZ) axis is perpendicular to it. BP is known for having strong linear dichroism, where light absorption along the AC direction is considerably larger than that along the ZZ direction. Due to the anisotropic absorption coefficient, the crystal axis can be easily determined from the polarized transmittance. Polarization-resolved transmission at a wavelength of 780 nm was performing by rotating a linearly polarized light with a half-wave plate. The result in Figure 1c exhibits a periodic transmission modulation with a period of 180, enabling us to clarify the crystal orientation of the AC and ZZ axis to be along the polarization of the minimum and maximum transmittance, respectively. 

In order to study the dynamics of lattice vibrations in BP, we performed time-resolved pump-probe experiments using ultrashort optical pulses from a Ti: sapphire laser with a repetition rate of 80 MHz and a pulse duration of about 50 fs. A beam splitter was used to divide the pulses into a pump beam of 4 mW and a weaker probe beam. In order to adjust the pump or probe polarization independently, we placed a combination of a linear polarizer and a half-wave plate in the path of each beam. When the pump pulse excites the BP crystal, electron and hole carriers are instantaneously created at the conduction and valence bands, and an abrupt change can be induced in the equilibrium coordinate of the lattice. The crystal lattice moves accordingly, leading to macroscopic oscillatory motions or CP oscillations of some specific modes. This CP generation mechanism available in opaque media is known as DECP (displacive excitation of CP) [33]. Because a distorted lattice influences the optical properties of the material, CP oscillation can be visualized by acquiring the time-resolved optical transmittance of the probe pulse through the sample under study.

To implement the time delay between the pump and probe optical pulses, we placed a retroreflector mirror for the pump pulse on top of a mechanical shaker moving at a repetition rate of 13 Hz. A × 20 objective lens was used to focus the pump and probe pulses into an overlapped spot of ≈ 4 µm diameter on the BP flake. In order to study the in-plane anisotropy of BP, we measured the transient transmittance (TT) as a function of time delay with rotations of both pump and probe polarizations. By accumulating TT signals repeatedly, we could reduce the noise and obtain high-quality signals sufficient to visualize the lattice vibrations in multilayer BP flakes.

## 3. Results and Discussion

**Figure 2:**
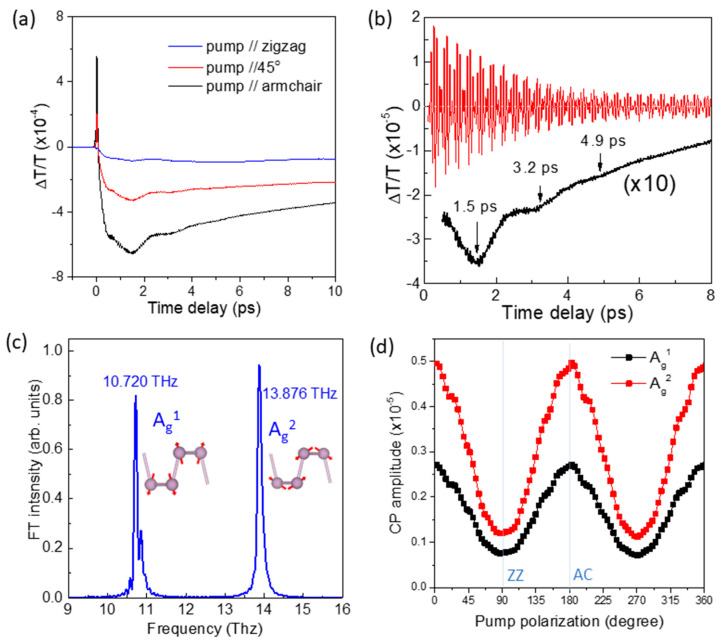
(**a**) Photoinduced TT changes of sample A at different pump polarizations with the probe polarization fixed along the AC axis. (**b**) Transmission modulation due to CP oscillations for the AC polarized pump beam (black line). The red line indicates CP oscillations after subtracting the slowly varying parts from the TT signal. (**c**) Fourier transformation spectrum of the time-resolved TT oscillation in Figure 2b. (**d**) Pump-polarization resolved CP amplitudes of the A_g_^1^ and A_g_^2^ modes.

With the pump-probe method, BP crystal can be investigated in both the excitation and detection processes, as related to pump and probe polarization, respectively. First, we discuss the pump polarization dependence of sample A obtained at a wavelength of 780 nm. Figure 2a shows photoinduced TT changes at different pump polarizations with the probe polarization fixed along the AC axis. Because the excited carrier density varies according to the linear dichroism of BP, the TT signal is strongest when the pump polarization is along the AC axis. Here, we do not pay much attention to the shape of the TT evolution as a function of time delay, but just note that the shape is almost the same for all pump polarizations.

When a material is excited by an optical pulse with a duration shorter than a particular phonon period, CP oscillation can be generated. We find that the TT signal in Figure 2a accompanying the high-frequency modulations originates from CP oscillations. The red line in Figure 2b, obtained by subtracting the slowly varying parts from the TT signal, reveals CP oscillations clearly. Here, the time-domain beating is a signature of multiple phonon modes. The Fourier transformation (FT) spectrum in Figure 2c indicates that the CP signal consists of two dominant frequencies, 10.72 and 13.88 THz, which correspond to the A_g_^1^ and A_g_^2^ symmetry optical phonons of orthorhombic BP crystal [18,19,20,21,26,27]. As shown in Figure 2c, the phosphorous atoms move largely in the out-of-plane (in-plane) direction within the A_g_^1^ (A_g_^2^) mode. We need to mention that the B_2g_ mode is as strong as the A_g_^1^ or A_g_^2^ mode in Raman scattering spectroscopy [19], but it is not observed in the CP signal here possibly because of the distinct crystal symmetry of BP that leads to negligible diagonal components in the Raman tensor [34]. As indicated by the black arrows in Figure 2b, we also notice a low-frequency signal with a period of about 1.7 ps, which possibly originates from the inter-layer lattice vibration of multilayer BP crystals [35,36].

Figure 2d shows the A_g_^1^ and A_g_^2^ mode CP amplitudes as functions of the pump polarization. The change in periodic amplitude with pump polarization for both modes is consistent with the linear dichroism in Figure 1c and the anisotropic TT strength in Figure 2a, which confirms that the impulse generating CP oscillation is proportional to the photoexcited carrier density.

**Figure 3:**
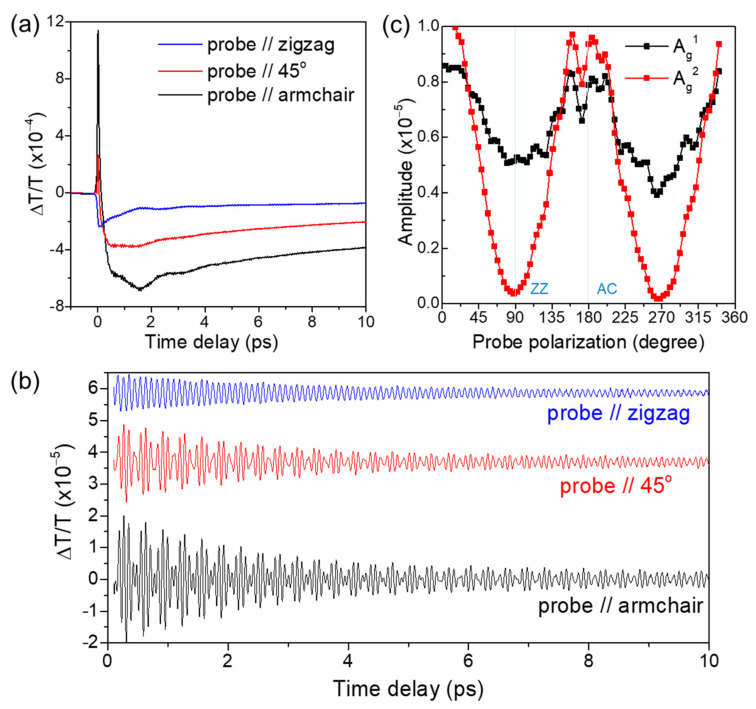
(**a**) Time-resolved TT signals of sample A at different probe polarizations with the pump polarization fixed along the AC axis. (**b**) CP oscillations extracted from the TT signals at different probe polarizations. (**c**) A_g_^1^ and A_g_^2^ mode CP amplitudes as a function of probe polarization.

Next, we discuss the probe polarization dependence of sample A at the same wavelength of 780 nm. Figure 3a shows time-resolved TT signals at different probe polarizations, with the pump polarization fixed along the AC axis. It was shown in Figure 2a that the temporal evolution of the TT moderately changes its shape under pump polarization variation. However, in the probe polarization dependence in Figure 3a, the difference in TT shape by probe polarization is more pronounced at small time delays, less than 2 ps. The CP oscillations in Figure 3b exhibit a strong probe polarization dependent temporal shape change as well: while the simple sinusoidal signal for the ZZ polarization, presumably from a single oscillator, decays monotonically, time-domain beating is prominent for the AC polarization. Figure 3c shows the amplitudes of the A_g_^1^ and A_g_^2^ modes as functions of the probe polarization. We note that the probe polarization dependence is not the same for the two A_g_ modes, with the A_g_^2^ mode changing more drastically with probe polarization. Because the pump pulse plays a role only in the generation process, the probe polarization dependence must be explained within the detection process.

Macroscopic lattice distortions by CPs can modulate the optical constants of the material. For a lattice distortion of a phonon mode *m*, *Q_m_*(*t*), the induced time-dependent optical susceptibility modulation, can be described as *x_i_*(*t*) = *x*_*i*0_ + (*∂x_i_*)/(*∂Q_m_*) × *Q_m_*(*t*), where *x*_*i*0_ is the susceptibility without distortion and *i* is the polarization. In the case of the A_g_ phonons in this study, we can use the reduced Raman tensor (*∂x_i_*)/(*∂Q_m_*) instead of the more general Raman tensor because the off-diagonal components are zero in the Raman tensor [18]. The transmission will then be modulated following the relation ∆*T_i_* = (*∂T_i_*)/(*∂x_i_*) × (*∂x_i_*)/(*∂Q_m_*) × *Q_m_*(*t*). To understand the probe polarization dependence, we need to consider both terms (*∂T_i_*)/(*∂x_i_*) and (*∂x_i_*)/(*∂Q_m_*), where the former term accounts for the transmission modulation due to the susceptibility change. The distinct behavior of the probe polarization dependence between the A_g_^1^ and A_g_^2^ modes in Figure 3c demonstrates that the polarization dependence of the (*∂x_i_*)/(*∂Q_m_*) term is not the same for the two phonon modes. We note that this observation is consistent with the reported different Raman tensor coefficients of the two A_g_ phonons [18,21]. 

**Figure 4:**
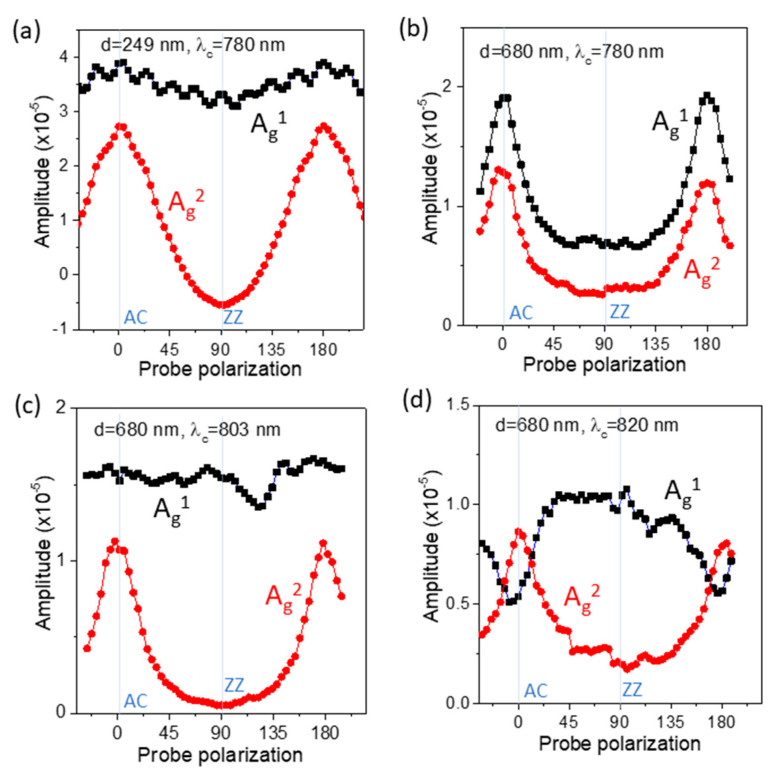
(**a**,**b**) Probe polarization dependence of the A_g_^1^ and A_g_^2^ mode CP amplitudes for BP flake thicknesses of 249 and 680 nm at a laser wavelength of 780 nm. (**c**,**d**) Those at laser wavelengths of 803 and 820 nm for the 680 nm thick BP flake.

In order to gain further insight into the anisotropic behaviors in the detection process, we explored the probe polarization dependence at other laser wavelengths and BP flake thicknesses. In Figure 4a,b, we show the probe polarization-dependent phonon amplitudes for BP flakes with thicknesses of 249 nm and 680 nm, sample B and sample C, respectively, at the same laser wavelength of 780 nm. Figure 4c and 4d show those at laser wavelengths of 803 nm and 820 nm from the 680 nm thick BP flake, respectively. In contrast to few-layer BP samples, interference between the multiple light reflections becomes important for rather thick samples and can exhibit strong dependence on the laser wavelength and the sample thickness. The obtained results show distinct probe polarization dependences for each combination of sample thickness and laser wavelength. Here, we need to mention that the polarized Raman scattering in BP has previously been reported to be similarly dependent on the incident wavelength and the sample thickness, which was explained by the interference effect of the incident and scattered light [18].

Because of the linear dichroism and the in-plane birefringence of BP, the polarization dependence due to the interference effect from the multiple reflections will vary with the wavelength and sample thickness. In the CP detection process, the interference effect must be incorporated into the term (*∂T_i_*)/(*∂x_i_*), which will vary with the sample thickness and wavelength as well as the polarization. Thus, the probe polarization dependence of the A_g_^1^ and A_g_^2^ modes results from the combined effect of the mode-dependent (*∂x_i_*)/(*∂Q_m_*) term and the above-mentioned (*∂T_i_*)/(*∂x_i_*) term. It is notable that while Raman scattering measurements of light intensity are insensitive to the phase of the phonon oscillation, the CP signal in this study can enable us to identify the initial phase of phonon oscillations.

As an example, the negative amplitude at the ZZ polarization of the A_g_^2^ mode in Figure 4a is directly obtained from the phase information of the CP signal. We reserve a detailed interpretation on the probe dependent A_g_^1^ and A_g_^2^ amplitudes at each specific wavelength and sample thickness for future work supported by theoretical calculations.

**Figure 5:**
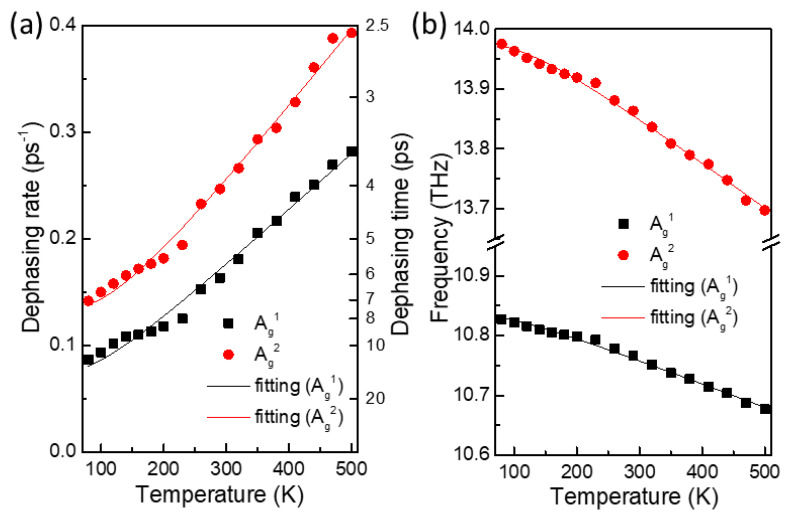
(**a**) Dephasing rate and (**b**) frequency of the A_g_^1^ and A_g_^2^ mode CP oscillations as a function of temperature. The solid lines are fittings based on the anharmonic phonon decay process into two half-frequency acoustic phonons.

One definite advantage of the time-resolved CP detection process in this work is the capability to visualize the phonon decay process. In order to study the phonon decay mechanism, we measured the CP oscillations of a 195 nm thick BP flake with varying temperature from 80 to 500 K at a wavelength of 780 nm. Figure 5a,b show the decay rates and the phonon frequencies of the A_g_^1^ and A_g_^2^ modes as a function of sample temperature. During the temperature increase from 80 to 500 K, the dephasing time quickened from 11.5 ps to 3.5 ps and from 7.0 ps to 2.5 ps for the A_g_^1^ and A_g_^2^ mode, respectively. According to the anharmonic phonon decay model, an optical phonon can be split into two or more low-frequency phonons while satisfying energy and momentum conservation, and this process is accelerated at elevated temperatures [37]. Because the most plausible and frequently observed anharmonic decay channel of optical phonons is that into two acoustic phonons of equal frequency and opposite wave vectors, we tried this decay channel to fit the temperature dependence of the decay rates and frequencies [28,38]. Here, the fitting functions for the dephasing rate (Γ) and the frequency (Ω) are given by Γ = Γ_0_ + Γ_anh_ × [1 + 2/(exp(ħΩ/2k_B_T) − 1)] and Ω = Ω_0_ + Ω_anh_ × [1 + 2/(exp(ħΩ/2k_B_T) − 1)], where Γ_anh_ and Ω_anh_ are proportional constants and Γ_0_ and Ω_0_ are background contributions to the dephasing rate and frequency, respectively. As shown with the solid lines in Figure 5a,b, the fitting is satisfactory for both A_g_^1^ and A_g_^2^ modes, which strongly suggests that the A_g_^1^ and A_g_^2^ phonons excited in BP crystal undergo a temperature-dependent anharmonic decay into two half-frequency acoustic phonons. We note that depending on the crystal purity or charge doping, the phonon decay can be accelerated due to such effects as defect scattering or electron–phonon interaction. Nevertheless, the anharmonic phonon decay process will universally apply to BP crystals of moderate thickness, in which the phonon dispersion is more or less the same as bulk BP. 

## 4. Conclusions

By performing time-resolved TT measurements, we investigated the in-plane anisotropies of BP crystal provided in the generation and detection process of coherent A_g_^1^ and A_g_^2^ phonons. The CP amplitudes generated by the pump pulse were found to be proportional to the excited carrier density, and the pump polarization dependence confirmed the linear dichroism of BP. The probe polarization dependence though, which behaves differently according to the laser wavelength and the sample thickness, needs to be explained by the combined effects of the phonon-induced optical susceptibility and the interference effect, which themselves are both dependent on the probe polarization. We also demonstrated the temperature-dependent anharmonic decay process of the A_g_^1^ and A_g_^2^ phonons. We expect that the anisotropic characteristics revealed in the excitation and detection of CPs will facilitate polarization-sensitive novel applications based on BP crystal and deepen our understanding of the optoelectronic properties of anisotropic 2D materials.

## Data Availability

Not applicable.

## References

[1] Xia F., Wang H., Jia Y. (2014). Rediscovering black phosphorus as an anisotropic layered material for optoelectronics and electronics. Nat. Commun..

[2] Gusmao R., Sofer Z., Pumera M. (2017). Black phosphorus rediscovered: From bulk material to monolayers. Angew. Chem. Int. Ed. Engl..

[3] Anju S., Ashtami J., Mohanan P.V. (2019). Black phosphorus, a prospective graphene substitute for biomedical applications. Mater. Sci. Eng. C Mater. Biol. Appl..

[4] Lee T.H., Kim S.Y., Jang H.W. (2016). Black phosphorus: Critical review and potential for water splitting photocatalyst. Nanomaterials.

[5] Li L., Han W., Pi L., Niu P., Han J., Wang C., Su B., Li H., Xiong J., Bando Y. (2019). Emerging in-plane anisotropic two-dimensional materials. InfoMat.

[6] Castellanos-Gomez A. (2015). Black phosphorus: Narrow gap, wide applications. J. Phys. Chem. Lett..

[7] Ling X., Wang H., Huang S., Xia F., Dresselhaus M.S. (2015). The renaissance of black phosphorus. Proc. Natl. Acad. Sci. USA.

[8] Tian H., Guo Q., Xie Y., Zhao H., Li C., Cha J.J., Xia F., Wang H. (2016). Anisotropic black phosphorus synaptic device for neuromorphic applications. Adv. Mater..

[9] Yuan H., Liu X., Afshinmanesh F., Li W., Xu G., Sun J., Lian B., Curto A.G., Ye G., Hikita Y. (2015). Polarization-sensitive broadband photodetector using a black phosphorus vertical p-n junction. Nat. Nanotechnol..

[10] Engel M., Steiner M., Avouris P. (2014). Black phosphorus photodetector for multispectral, high-resolution imaging. Nano Lett..

[11] Chen X., Lu X., Deng B., Sinai O., Shao Y., Li C., Yuan S., Tran V., Watanabe K., Taniguchi T. (2017). Widely tunable black phosphorus mid-infrared photodetector. Nat. Commun..

[12] Wang X., Jones A.M., Seyler K.L., Tran V., Jia Y., Zhao H., Wang H., Yang L., Xu X., Xia F. (2015). Highly anisotropic and robust excitons in monolayer black phosphorus. Nat. Nanotechnol..

[13] Schuster R., Trinckauf J., Habenicht C., Knupfer M., Buchner B. (2015). Anisotropic particle-hole excitations in black phosphorus. Phys. Rev. Lett..

[14] Mao N., Tang J., Xie L., Wu J., Han B., Lin J., Deng S., Ji W., Xu H., Liu K. (2016). Optical anisotropy of black phosphorus in the visible regime. J. Am. Chem. Soc..

[15] Lee S., Yang F., Suh J., Yang S., Lee Y., Li G., Sung Choe H., Suslu A., Chen Y., Ko C. (2015). Anisotropic in-plane thermal conductivity of black phosphorus nanoribbons at temperatures higher than 100 K. Nat. Commun..

[16] Tao J., Shen W., Wu S., Liu L., Feng Z., Wang C., Hu C., Yao P., Zhang H., Pang W. (2015). Mechanical and electrical anisotropy of few-layer black phosphorus. ACS Nano.

[17] Luo Z., Maassen J., Deng Y., Du Y., Garrelts R.P., Lundstrom M.S., Ye P.D., Xu X. (2015). Anisotropic in-plane thermal conductivity observed in few-layer black phosphorus. Nat. Commun..

[18] Kim J., Lee J.U., Lee J., Park H.J., Lee Z., Lee C., Cheong H. (2015). Anomalous polarization dependence of Raman scattering and crystallographic orientation of black phosphorus. Nanoscale.

[19] Zhang S., Yang J., Xu R., Wang F., Li W., Ghufran M., Zhang Y.-W., Yu Z., Zhang G., Qin Q. (2014). Extraordinary photoluminescence and strong temperature/angle-dependent Raman responses in few-layer phosphorene. ACS Nano.

[20] Sugai S., Shirotani I. (1985). Raman and infrared reflection spectroscopy in black phosphorus. Solid State Commun..

[21] Ribeiro H.B., Pimenta M.A., De Matos C.J., Moreira R.L., Rodin A.S., Zapata J.D., De Souza E.A., Castro Neto A.H. (2015). Unusual angular dependence of the Raman response in black phosphorus. ACS Nano.

[22] Wu J., Mao N., Xie L., Xu H., Zhang J. (2015). Identifying the crystalline orientation of black phosphorus using angle-resolved polarized Raman spectroscopy. Angew. Chem. Int. Ed. Engl..

[23] Phaneuf-L’Heureux A.L., Favron A., Germain J.F., Lavoie P., Desjardins P., Leonelli R., Martel R., Francoeur S. (2016). Polari-zation-resolved raman study of bulk-like and davydov-induced vibrational modes of exfoliated black phosphorus. Nano Lett..

[24] Ling X., Huang S., Hasdeo E.H., Liang L., Parkin W.M., Tatsumi Y., Nugraha A.R., Puretzky A.A., Das P.M., Sumpter B.G. (2016). Anisotropic electron-photon and elec-tron-phonon interactions in black phosphorus. Nano Lett..

[25] Mao N., Wu J., Han B., Lin J., Tong L., Zhang J. (2016). Birefringence-directed raman selection rules in 2D black phosphorus crystals. Small.

[26] Li R., Shang Y., Xing H., Wang X., Sun M., Qiu W. (2020). Orientation identification of the black phosphorus with different thickness based on B_2g_ mode using a micro-raman spectroscope under a nonanalyzer configuration. Materials.

[27] Li R., Sun M., Shang Y., Xing H., Wang X., Qiu W. (2021). Crystalline orientation identification of multilayer black phosphorus based on the A_g_^1^ and A_g_^2^ raman modes for an orthogonally polarized configuration. J. Phys. Chem. C.

[28] Jeong T.Y., Jin B.M., Rhim S.H., Debbichi L., Park J., Jang Y.D., Lee H.R., Chae D.-H., Lee D., Kim Y.-H. (2016). Coherent lattice vibrations in mono-and few-layer WSe2. ACS Nano.

[29] Dekorsy T., Cho G.C., Kurz H. (2000). Coherent phonons in condensed media. Light Scatt. Solids VIII.

[30] Cho G., Kütt W., Kurz H. (1990). Subpicosecond time-resolved coherent-phonon oscillations in GaAs. Phys. Rev. Lett..

[31] Jeong T.-Y., Bae S., Lee S.-Y., Jung S., Kim Y.-H., Yee K.-J. (2020). Valley depolarization in monolayer transition-metal dichalcogenides with zone-corner acoustic phonons. Nanoscale.

[32] Vialla F., Del Fatti N. (2020). Time-domain investigations of coherent phonons in van der waals thin films. Nanomaterials.

[33] Zeiger H., Vidal J., Cheng T., Ippen E., Dresselhaus G., Dresselhaus M. (1992). Theory for displacive excitation of coherent phonons. Phys. Rev. B.

[34] Norimatsu K., Hada M., Yamamoto S., Sasagawa T., Kitajima M., Kayanuma Y., Nakamura K.G. (2015). Dynamics of all the Raman-active coherent phonons in Sb_2_Te_3_ revealed via transient reflectivity. J. Appl. Phys..

[35] Luo X., Lu X., Koon G.K., Castro Neto A.H., Ozyilmaz B., Xiong Q., Quek S.Y. (2015). Large frequency change with thickness in interlayer breathing mode—Significant interlayer interactions in few layer black phosphorus. Nano Lett..

[36] Miao X., Zhang G., Wang F., Yan H., Ji M. (2018). Layer-dependent ultrafast carrier and coherent phonon dynamics in black phosphorus. Nano Lett..

[37] Klemens P. (1966). Anharmonic decay of optical phonons. Phys. Rev..

[38] Song D.Y., Nikishin S.A., Holtz M., Soukhoveev V., Usikov A., Dmitriev V. (2007). Decay of zone-center phonons in GaN with A1, E1, and E2 symmetries. J. Appl. Phys..

